# Midwifery

**Published:** 1837-04

**Authors:** 


### MIDWIFERY.

*Case of Extra-Uterine Pregnancy.* By William Bell, m.d.

On the evening of the 11th of June, I was requested to visit Dorothy Morris, an apprentice on Golden-Grove Estate, forty years of age, and the mother of five children. For a considerable period (nearly four years, according to her own statement), she had occasionally experienced slight abdominal uneasiness, with the feeling of a hard swelling in the right hypochondrium; but the pain being inconsiderable and not constant, she continued at work until within the last three months, nor had she found it necessary to make any application for advice before the present time. On being questioned, she stated that the catamenia, with one exception, had not been interrupted, and that the appetite and general health continued unimpaired. When I saw her, there was a dark-coloured fœtid mass projecting through an opening in the abdominal parietes, about two inches under, and a little to the right side of the umbilicus, which was found to be the remains, or rather the whole, of a fœtal cranium, surrounded by a plentiful discharge of very offensive putrid matter. As the expulsive efforts appeared ineffectual, it was removed, and observed to be an entire fœtus, of the size apparently of five months. No remnants of the placenta or cord could be discovered; but the midwife remarked that something like the former had been discharged previous to my visit. The abscess was carefully washed with tepid water; a compress of lint applied and secured by a bandage. Quiet, and the supine posture were recommended. As the patient had no febrile symptoms (pulse eighty), and complained of no uneasiness, medicine was not considered necessary. The fœtus was washed with rum, and its different members were readily distinguished; but it would amount to conjecture only, were I, from that circumstance, to draw any inference as regards the length of time it had remained in the abdomen. The statement of the mother on this point, I am inclined, however, to believe, is nearly correct. The result of the case may be given in a few words:—The patient suffered from no symptom of fever, inflammation, or any other complaint during the whole course of the cure of the abscess.

Her appetite could scarcely be satisfied, and, on the 14th of July, she was walking in her garden, the opening nearly closed, with a slight gleety discharge: the poultice, which had been the only application, was now laid aside, and a more stimulating treatment adopted. She is now perfectly recovered, without having experienced an unfavorable symptom, and without having required a dose of medicine. There is one circumstance deserving of notice, which is the absence of all constitutional disorder, the patient having suffered from no symptom requiring the aid of medicine. In similar accidents, the greater proportion of which (so far as recorded) have occurred to European practitioners, such an immunity from suffering I believe to have been but rarely met with; and it affords a favorable opportunity of hazarding an opinion, which, from other causes, I am inclined to entertain, that the African race in general seem to suffer less constitutionally from serious accidents and operations than their otherwise more favoured and civilized brethren.

*Jamaica Physical Journal, December*, 1835.

---

*On the Reposition of the Umbilical Cord.* By Dr. MICHAELIS.

THIS is a continuation of the paper we noticed in our second Number, (April, 1836,) p. 588: it contains seven additional cases of great interest, which show the importance of the plan recommended by Dr. M. For an explanation of the conditions requisite to ensure successful reposition of the cord, and its being retained above the presenting part, we must refer to the above-mentioned notice. We will now select two or three of these cases, in further illustration of the subject.

CASE. Dr. Michaelis had turned the child in a former labour, on account of faulty position (arm or shoulder), on the 11th of January, 1835. He found the membranes ruptured, the abdomen of a very irregular configuration, and the whole left arm, together with a large coil of the slowly pulsating funis, in the vagina. The head could be reached at the brim of the pelvis on the left side. He returned the arm without difficulty, but could not succeed in replacing the funis, as the uterus evinced no disposition to contract round the head, which now presented to the pelvis more favorably. Not choosing to give up the hopes of succeeding, he determined to wait for greater uterine activity. In this, however, he was disappointed, for a knee presented near to the head; and, finding there was no chance of success in replacing the funis with so inactive a uterus, he brought down the knee, and quickly delivered the woman of a living child. The funis was actually fifty-three inches long, and twice round the neck.

Although his attempts to return the funis were unsuccessful, the case shows how necessary it is that there should be a certain degree of uterine contraction round the head of the child, to ensure the cord being retained above it.

CASE. A primipara had had pains since the morning. Dr. Michaelis examined at noon. The abdomen was strongly inclined to the left side; a narrow bladder of membranes had descended into the vagina through the os uteri, which was nearly fully dilated. The cord was felt beating, with strong and rapid pulsations, within the membranes; and the head was reached with difficulty above the brim. As the patient was very sensitive during examination, Dr. M. expected he should find it necessary to use the catheter in replacing the cord; but, on introducing half of his hand, to ascertain the position of the head, he found he could do it without. As the cord hung down from the left side, he easily returned it in this direction, and the annular contraction of the uterus followed immediately, although not very completely, from the quantity of liquor amnii. He therefore ruptured the membranes, and allowed the liquor amnii to escape slowly. The posterior fontanelle now descended at the left side of the brim, but the cord was still close by the occiput, although above the ring of uterine contraction. In sixteen hours after the reposition of the cord, a healthy living child was born. The cord was tight round the umbilicus, and measured twenty-seven inches.

CASE. It was the patient's second labour, and commenced in the morning. At noon, the os uteri was dilated; and, at half-past four, a considerable quantity of liquor amnii escaped. A coil of the cord, pulsating strongly, and about four inches long, hung down into the vagina on the right side. The abdomen was slightly pendulous, and the os uteri inclined to the right side. Dr. Michaelis introduced his left hand, and, gathering the coil upon the points of his fingers, carried it upwards along the hollow of the sacrum. The circular contraction of the inferior segment of the uterus had already taken place. He allowed the liquor amnii to flow off entirely, and supported the abdomen: the head immediately descended upon the os uteri, and entered the pelvis. In three hours after, the child was born alive: it weighed seven and a half pounds; the cord was tight round its neck.

*Neue Zeitschrift für Geburtskunde,* Vol. iv. 1836.

---

*Case of Delivery after Episioraphy.* By Dr. Platt, of Hamburg.

A. D. B., aged thirty-six, had borne a child eighteen years previously without any remarkable difficulty, and afterwards continued to enjoy good health for several years. In 1831, she became affected with "a bearing down," which in the course of half a year increased to a complete prolapsus of the uterus and vagina. She went into the general Hospital at Hamburg, where she was treated with pessaries of various shapes and sizes, but without advantage; the pessaries were either not retained *in situ,* or they produced ulcerations and excoriating discharges. Episioraphy was therefore performed by Dr. Fricke, and with success. By the cohesion of the labia, a fleshy bridge was formed, more than an inch in breadth, and sufficiently strong to prevent any further prolapse of the uterus. The patient married in 1834, and soon after became pregnant. Feeling a considerable degree of anxiety about her condition, she again applied to Dr. Fricke, who recommended her to the care of Dr. Platt. At the end of the eighth month of pregnancy, the state of the genitals were found as follows: The labia majora formed by their union an oval flattened protuberance of considerable extent, with two nearly circular openings, of which the lower was two inches, and the upper an inch and a half in diameter; the intervening fleshy bridge measured fully an inch and a quarter in breadth, and was about one-eighth of an inch in thickness. A confused mass, consisting of the prolapsed walls of the vagina, filled up both openings, and had been forced down by the pressure of the superincumbent parts, so that the lower opening, originally very small, but on which the pressure was chiefly directed, had become the larger of the two. In the other opening the nympha could be felt. By pressing up the external parts with a considerable degree of force, the head of the child could be felt at the entrance of the pelvis. The general state of the patient was favorable, except that she felt considerable anxiety about the issue of her labour.

On the evening of the 15th of May labour came on. Next day the os uteri was completely dilated, the waters came away, and the head could be felt lying in the first position. Some hours afterwards, in consequence of the labour becoming tedious, the forceps was applied through the posterior opening, and Dr. Platt, having brought down the head as far as the bridge, awaited the result. The pains now became very strong, and the edges of the opening were stretched to the utmost extent. To avoid a laceration of the parts, the amount of which it would be impossible to determine, Dr. Fricke made an incision with a button-pointed bistoury on each side of the opening to the depth of two lines, and subsequently another more anteriorly on the right side; whereupon the head, and a few moments afterwards the rest of the body, was born. The placenta came away soon after, and the rest of her confinement went on quite regularly. In the course of a few days, the surfaces of the incisions exhibiting a languid appearance, were touched occasionally with a mixture of balsam of Peru and tincture of myrrh. The patient was not able to leave her bed for several weeks, and subsequently used for a long time compresses dipped in a decoction of oak-bark, and applied with a T bandage. Under this plan of treatment the parts returned to their former state, and the patient still enjoys all the advantages derived from the original operation.

### *On the Use of the Plug in Cases of Placenta Prævia.* By Dr. ALBERT, of Wiesentheid.

[THREE cases are reported where the tampon was used to afford the os uteri time for sufficient dilatation. We are quite ready to allow Wigand the due merit of having written some admirable observations on this subject; still, however, we must not forget the original merit of Leroux. The value of the plug in profuse hemorrhage from placenta prævia, where the os uteri is rigid and but little dilated, has not been sufficiently estimated. Where it is made of proper materials, it may remain in the vagina, until, by the passage of the head into this canal, expulsive pains are induced, and further hemorrhage prevented.]

CASE. A robust, plethoric woman, mother of seven children, was attacked with slight hemorrhages from the vagina, which recurred every three or four days, from the latter end of the eighth to the middle of the tenth month of her pregnancy, when the discharge became much more profuse. The placenta was found centrally situated upon the os uteri, which was dilated to about the size of a shilling. Although she was not exhausted, yet, as her condition was not free from danger, Dr. A. prepared a plug of fine linen, which was oiled, and passed up the vagina against the os uteri; the vagina was also stuffed with charpie, and the whole secured by a proper bandage. The patient was desired to remain quiet in the supine posture, with her knees together, and she was left for some hours under the charge of a midwife, with proper instructions. After a considerable period, Dr. A. was again called; expulsive pains having come on, during which the plug produced much straining and tension. On removing it, the liquor amnii escaped, and a small quantity of blood in clots. The right edge of the placenta was detached, and hung down against the left side of the vagina. The os uteri was fully dilated, and the largest circumference of the head had passed through it. The child was expelled in about half an hour; the placenta followed immediately, and nothing occurred to disturb her getting up. The child was alive and active.

[We do not approve of the plug used by Dr. A., although sanctioned by Mr. Burns's authority. It is far inferior to the sponge plug, as recommended by Dr. Dewees. This is introduced and removed with far greater ease, and, being much softer, is retained in the vagina without inconvenience by the patient. Our objections to the linen plugs are illustrated in Dr. A.'s next case, where it required to be altered twice before it could be retained by the patient.]

*Neue Zeitschrift für Geburtskunde*, Vol. iv. No. 1 and 2. 1836.

---

### *On the Means of inducing Uterine Contraction.* By Dr. SCHNEIDER, of Fulda.

A REMEDY, which is generally sure to produce contraction in cases of obstinate uterine atony, is placing the new-born child to its mother's breast. We obtain this sanative effect by means of the well-known sympathy between the breast and uterus. The uterus contracts, and the flooding stops.

[This brief extract is taken from a paper entitled "Contributions from the Practice of an old Obstetrician;" and we notice it because we think the fact, the truth of which we have had repeated opportunities of proving, is not so generally known as it ought to be.] *Neue Zeitschrift für Geburtskunde*, Vol. iv. 1836.

---

### *Contributions to the Pathology of Puerperal Fever.* By Dr. A. BARTELS.

THESE observations are taken from cases seen in the Lying-in Hospital of Vienna. Dr. B. notices the fact of puerperal ulcers being contagious, and that they may easily be communicated to healthy patients, by using the same sponge, &c. This fact we have pointed out in our Review of Mr. Moore's work on Puerperal Fever, p. 488, and shown that, in some aggravated cases, the contagion may be communicated even to females *not* in the puerperal state. Dr. B. has quoted an exceedingly interesting observation by Professor Schönlein, who considers that the contagion of puerperal fever possesses the greatest analogy to that of hospital gangrene. He grounds this opinion on the remarkable similarity which he has observed between ulcers in the puerperal state and hospital gangrene. According to some investigations which he made some years ago at Würzburg, it would appear that the one could be developed from the other affection. When we compare the observations of Dr. John Thomson with these views,—viz. that hospital gangrene sometimes appears in the form of small pustules, without previous breach of surface,—their importance will be better seen.

*Neue Zeitschrift für Geburtskunde,* Vol. iv. 1836.

